# Supplementary material for: Decoding budget awareness: A multivariate analysis of generation Z undergraduates
Source: PLoS One. 2025 Aug 14;20(8):e0328742. doi: 10.1371/journal.pone.0328742 (PMC12352852; doi:10.1371/journal.pone.0328742)
Supplement: Attachment — Submitted filename: Review of Decoding Budget Awareness.docx [file pone.0328742.s001.docx]

**RESULT OF REVIEW**

Title: *Decoding Budget Awareness: A Multivariate Analysis of Generation Z Undergraduates*

Previously, I would like to thank you for assigning me to review this paper. I have read and scrutinized the article and found the author has presented an interesting paper on *Decoding Budget Awareness: A Multivariate Analysis of Generation Z Undergraduates*.

COMMENTS FROM REVIEWER

1. I express my highest appreciation to the author, who has made many improvements by incorporating various inputs from previous reviewers.
2. Article structure:

The Title, Abstract, Introduction, Literature Review, and Research Methodology groups consist of 3,594 words (43.58%) of the total paper. The Results and Discussion, Conclusion, and Recommendations groups consist of 2,749 words (33.33%) of the total paper. References consist of 1,904 words (23.09%) of the total paper. In my opinion, the composition of this paper is not proportional because the Core Section of the Paper (Results, Discussion, and Conclusion) is less than 50.00% of the total paper.

3. Originality

This study has a unique theme with the state budgeting theme for Generation Z undergraduate students in four Turkish state universities. The study's results may be a reference for other countries for budgeting for students in government universities.

4. Abstract

The author has compiled an abstract containing the objectives, methods, findings, implications, contributions, and recommendations accompanied by keywords representing the paper's discussions. However, the author has not included the background of this research.

5. Utilities of literature

The 78 references used in this work are all taken from journal journals. Of the 78 articles, 40 (51.28%) were published in 2018 or later (less than five years old), while 38 (48.72%) were published before 2018 (more than five years old). The paper's quality has been enhanced by the author's use of references from internationally indexed journal papers that are relevant to the topic under discussion. Seventy-five per cent of the references cited in some journals must be no more than five years old. The author might want to take this into account.

6. Methodology

The author uses a multivariate linear regression analysis method to separate independent and dependent variables. A survey of 3,972 undergraduate students enrolled in four Turkish state universities across all subject areas between December 2023 and April 2024 was used to gather data for the analysis. While the survey data becomes the conclusions reported in the Results and Discussion section, the author may hypothetically formulate the research method employed in this section.

7. Results and Discussions

The author of this section mixes findings and discussions. This mixing of findings and discussions makes it difficult for readers to distinguish which is the research findings data and which is the analysis results data. It is better to divide this section into two subsections, namely (sub) Results and (sub) Discussions. In the sub-results, the author can add a little about the respondent profile, followed by an analysis of the survey data, which can be presented in the form of tables and figures. Furthermore, in the sub-discussion, the author can discuss all the findings data (all tables and figures).

8. Conclusion

The author has developed a lengthy conclusion. However, it's important to keep in mind that the conclusion serves as both a summary of the research findings and an answer to the research questions that were posed and debated during the discussion. In the Conclusion and Recommendation section, the author should include a summary of the research findings and recommendations for how interested parties might use the study's findings.

9. Implications

The author emphasises how budgeting for Generation Z students has social and educational ramifications. Since the government is responsible for public monies, these ramifications force it to acknowledge the necessity of budgeting for Generation Z's education.

10. Quality of Communication

The paper's sections have been well-written by the author; however, keep in mind that readers like papers that are clear and cohesive.

**References**

1. Alkan, F. and Yildiz, F. (2016). “Research on the perception of budget of individuals in budgeting process of public institutions”. Electronic Journal of Vocational Colleges, October, 54-66.
2. Ansong, A. and Gyensare, M. (2012). “Determinants of university working-students financial literacy at the University of Cape Coast, Ghana”. International Journal of Business and Management, 7 (9), 126-133.
3. Araiza-Aguilar, J.A., Rojas-Valencia, M.N. and Aguilar-Vera, R.A. (2020). “Forecast generation model of municipal solid waste using multiple linear regression”. Global Journal of Environmental Science and Management, 6(1), 1–14.
4. Bartlett, J., Kotrlik, J.W. and Higgins, C.C. (2001). “Organizational research: Determining appropriate sample size in survey research”. Information Technology, Learning, and Performance Journal, 19(1), 43-50.
5. Beckker, K.D., Witte, K.T. and Campenhout, G.V. (2020). “The role of national culture in financial literacy: Cross-country evidence”. Journal of Consumer Affairs, 54, 912-930. DOI: 10.1111/joca.12306
6. Bendo, A. and Brovina, F. (2024). “A statistical model using multiple regression analysis to predict equilibrium and sway index”. Journal of Physical Education and Sport, 24(6), 1446– 1456.
7. Bowman, N.A. (2012). “Effect sizes and statistical methods for meta-analysis in higher education. Res High Educ, 53, 375–382. https://doi.org/10.1007/s11162-011-9232-5
8. Burhead, F. (1956). Government Budgeting. London: New York: John Wiley and Sons, Inc.
9. Chillakuri, B. (2020). “Understanding generation z expectations for effective on boarding”. Journal of Organizational Change Management, 33(7), 1277–1296.
10. Ciğerci, I. and Pehlivan, M. (2020). “Budget literacy: A study on Aegean region municipal council members”. Tax Report, 268, 191-217.
11. Cohen, J., Cohen, P., West, S. G. and Aiken, L. S. (2013). Applied multiple regression/correlation analysis for the behavioural sciences (3rd ed). UK: Routledge.
12. Davies, P. (2006). “Educating citizens for changing economies”. Journal of Curriculum Studies, 38 (1), 15–30.
13. Demirbaş, T. and Çetinkaya, Ö. (2018). The control and audit in public financial management. Ankara: Ekin Publishing.
14. Demirbilek, M. and Keser, S. (2022). “Leadership expectations of generation z teachers working in educational organizations”. Research in Educational Administration and Leadership, 7(1), 209–245.
15. Ebdon, C. and Franklin, A. L. (2006). “Citizen participation in budgeting theory”. Public Administration Review, 66(3), 437-447.
16. Eroğlu, E. and Kaynar, İ. (2023). “Budget literacy: An analysis of knowledge levels of city councilors relating to budgeting in Türkiye”. Amme Idaresi Journal, 56(3), 59-79.
17. Eroğlu, E., Aydemir, M., Tunç, G. and Gerçek, A. (2023). “An analysis of the relationship between municipalities and NGOs in terms of active participation in local budgeting in Türkiye. Sosyoekonomi, 31(56), 171-190.
18. Erten, P. (2019). "The attitudes of the z generation towards digital technology". Gümüşhane University Journal of Social Sciences, 10(1), 190–202.
19. Formanova, L., Madr, M., Andrlık, B. and Hrabalova, V. (2021). “Factors influencing the level of tax literacy of students of bachelor study programmes in economics at faculties of economics”. DANUBE, 12 (4), 308–323.
20. Freudenberga, B., Chardon, T., Brimble, M. and Isle, M. B. (2017). “Tax literacy of Australian small businesses”. HeınOnline, 1-46.
21. Giachino, C., Pucciarelli, F., Bollani, L., and Bonadonna, A. (2023). “Is generation z ready to fly into the space? The future of tourism is coming”. Futures, 145, 103064–103076.
22. Goncalves, S. (2014). “The effects of participatory budgeting on municipal expenditures and infant mortality in Brazil”. World Development, 53, 94-110.
23. Gümüş, N. (2020). "Investigation of generation Z consumers' purchasing decision making styles". Yaşar University E-Journal, 15(58), 381–396.
24. Güngör Göksu, G. (2020). “Citizen budget awareness and analysis of factors affecting the increase in budget awareness”. Dumlupinar University Journal of Social Sciences, 66, 126-146.
25. Güven, T. M. and Etçi, H. (2022). "Determining the technological competencies of generation Z against unemployment”. Journal of Economics and Management Research, 11(2), 53–72.
26. Hair, J. F., Willian C. B., Barry J. B. and Rolph E. A. (2010). Multivariate data analysis. USA: Pearson.
27. Hayes, A. F. (2017). Introduction to mediation, moderation, and conditional process analysis: A regression-based approach (2nd ed.). USA: Guilford Publications.
29. Hidayat, R. and Tauhid, M.S. (2022). “Co-learning on budget literacy and expenditure tracking of the village development program”. CARADDE: Jurnal Pengabdian Kepada Masyarakat, 5(2), 236-343. Doi: https://doi.org/10.31960/caradde.v5i2.1560
30. Jeger, M., Sušanj, Z. and Mijoč, J. (2014). “Entrepreneurial intention modelling using hierarchical multiple regression”. Croatian Operational Research Review, 5, 361–373.
31. Kaderli, Y. and Altinkan, D. (2022). “A quantitative research on the relationship between economic literacy and budget behaviour in youth”. Nazilli Faculty of Economics and Administrative Sciences Journal, 3(2), 67-83.
32. Kadre, S. and Konasani, V. R. (2015). Practical business analytics using SAS: A hands-on guide. USA: Apress.
33. Karadoğan, A. (2019). "Generation z and the teaching profession". Ağri Ibrahim Çeçen University Social Sciences Institute Journal, 5(2), 9–41.
34. Karatzimas, S. (2024). “Citizens’ perceptions on public finance literacy: Familiarity, importance, actual use and proposed learning objectives”. Teaching Public Administration, 42(1), 42-59.
35. Kayalidere, G., and Çakir, S. (2018). “Participatory budgeting and perception of participatory budget in city councils: The case of city council of Manisa Yunusemre municipality”. Management and Economics: Journal of Celal Bayar University Faculty of Economics and Administrative Sciences, 25 (3), 941-960.
36. Khotami, M. (2017). “The concept of accountability in good governance”. Advances in Social Science, Education and Humanities Research (ASSEHR),163, 30-33.
37. Killins, R.N. (2017). “The financial literacy of generation y and the influence that personality traits have on financial knowledge: Evidence from Canada”. Financial Services Review, 26(2). 143–165. https://doi.org/10.61190/fsr.v26i2.3305
38. Kim, N.W., Jung, J., Ko, E.Y., Han, S., Lee, C.W., Kim, J. and Kim, J. (2016). "Budget map: Engaging taxpayers in the issue-driven classification of a government budget”. Proceedings of the 19th ACM Conference on Computer-Supported Cooperative Work and Social Computing, 1028–1039.
39. Küçükaycan, D. and Güngör Göksu, G. (2021). “The effects of globalization on Turkish public financial management and budget process”. In Handbook of Research on Institutional, Economic, and Social Impacts of Globalization and Liberalization (pp. 273–286). USA: IGI Global.
40. Kupec, V. (2017). “Marketing information and its impact on generation Z”. Megatrendy a Médiá, 4(1), 264–278.
41. Lee, J., Kim, S. and Lee, J. (2022). "Public vs. public: Balancing the competing public values of participatory budgeting". Public Administration Quarterly, 46(1), 39–66.
42. Lin, X., Bruhn, A. and William, J. (2019). “Extending financial literacy to insurance literacy: A survey approach”. Accounting & Finance, 59, 685–713.
43. Lon, K.W., Khan, M.R.B., Lean, C.P. and Purnshatman, T. (2024). “Application of regression analysis in forecasting mobile broadband subscriptions growth for Malaysia”. Malaysian Journal of Business, Economics and Management, 3(1), 81–87.
44. Lyngdoh, T., El-Manstrly, D. and Jeesha, K. (2023). “Social isolation and social anxiety as drivers of generation Z’s willingness to share personal information on social media”. Psychology & Marketing, 40(1), 5–26.
45. Machova, R., Seben, Z. and Kutna, A. (2019). “Generation z and y versus tax literacy in the 21st Century”. Journal of Applied Economic Sciences, 2(64), 433-439.
46. Masud, H., Pfeil, H., Agarwal, S. and Briseno, G.A. (2017). International practices to promote budget literacy key findings and lessons learned, international bank for reconstruction and development . A World Bank study Washington, D.C.: World Bank Group.
47. McBeth, M.K. (2022). “Coddled or engaged? Teaching political tolerance to generation z students”. Journal of Political Science Education, 18(4), 438–454.
48. Mládková, L.(2017). "Learning habits of generation Z students." In European Conference on Knowledge Management. Academic Conferences International Limited, 2017.
49. Mulgan, R. (2000). “Accountability: An ever-expanding concept?”. Public Administration, 78(3), 555-573.
50. Neuby, B. L. (1997). “On the lack of a budget theory”. Public Administration Quarterly, 21(2), 131-142.
51. Nichita, R.A. (2015). “Knowledge is power. Improving tax compliance by means of boosting tax literacy”. Annals of Faculty of Economics, 1(1), 770-774.
52. Nikonova, E.N. and Shavaleeva, C.M. (2016). “Enhancement of public fiscal competence as educational process tendency in Russia”. SHS Web of Conferences, 26, 01045.
53. Norton, A. and Elson, D. (2002). What’s behind the Budget? Politics, rights and accountability in the Budget Process. London: ODI.
54. Park, J., Butler, J.S. and Petrovsky, N. (2023). “Understanding public participation as a mechanism affecting government fiscal out comes: Theory and evidence from participatory budgeting”. Journal of Public Administration Research and Theory, 33(2), 375-389. https://doi.org/10.1093/jopart/muac025
55. Republic of Türkiye Ministry of Treasure and Finance (2022). 2022 Citizen Final Account Report. Access 09 September 2024. https://ms.hmb.gov.tr/uploads/sites/3/2024/02/Vatandas- Kesin-Hesap-Raporu-2022.pdf
56. Republic of Türkiye Presidency of Strategy and Budget (2024). 2022 Citizen's Budget Guide. Access 09 September 2024. https:/[/www.sbb.gov.tr/wp-content/uploads/2024/01/2024-](http://www.sbb.gov.tr/wp-content/uploads/2024/01/2024-) VatandasinButce-Rehberi.pdf
57. Rubin, I.S. (1990). “Budget theory and budget practice: How good the fit?”. Public Administration Review, 50(2), 179-189.
58. Sapankaya, F.Y. and Tandircioğlu, H. (2022). “Youth's perception of the power of purse: A evaluation of students of Manisa Celal Bayar University Faculty of economics and administrative sciences”. Journal of Management and Economics, 29(2), 297-317.
59. Schick, A. (1966). “The road to PPB: The stages of budget reform”. Public Administration Review, 243-258.
60. Seçgin, Y. and Özyer, K. (2016). “The mediating role of empowerment on the affect of locus of control onto the organizational commitment: A survey on four and five stars hotel in the city centre of Ankara”. International Journal of Eurasia Social Sciences, 7(22), 249-277.
61. Selen, U. and Tarhan, A. B. (2015). “Power of the purse perception of voters in Türkiye and affecting factors”. Journal of Life Economics, 2(2), 1-18.
62. Sharma, G. (2017). “Pros and cons of different sampling techniques”. International Journal of Applied Research, 3(7): 749-752.
63. Singh, M., Ghai, S., Mishra, A.K., and Goyal, N. (2024). “Analysis of EAC using multiple regression and conditional process: A statistical approach”. Journal of Reliability and Statistical Studies, 17(1), 109–136.
64. Tabachnick, B.G. and Fidell, L.S. (2012). A practical approach to using multivariate analyses. Boston: Pearson.
65. Tekin, E. (2020). "The effect of COVID-19 anxiety on motivation: An investigation on generation Z". Electronic Turkish Studies, 15(4), 1129-1145.
66. Thomas, G. (2022). "Corporate social responsibility as a sustainable business practice: A study among generation Z customers of Indian luxury hotels”. Sustainability, 14(24), 16813. <https://doi.org/10.3390/su142416813>
67. Tomaskova, H., Mohelska, H. and Nemcova, Z. (2011). “Issues of financial literacy education”. Procedia-Social and Behavioral Sciences, 28, 365-369.
68. Touchton, M. and Wampler, B. (2014). “Improving social well-being through new democratic institutions”. Comparative Political Studies 47(10): 1442–69.
69. Tsurkan, M.V. (2017). "Analysis of regional participatory budgeting practices in the framework of local initiatives support program”. FinansovyjŽhurnal—Financial Journal, 3, 119–130.
70. Türegün, F.B., Gerçek, A., Gerger, G.C., Benk, S., Küçüksüleymanoğlu, E.R., Serbes, H., and Ateş, M.S. (2021). “Türkiye’de üniversite öğrencilerinin vergi okuryazarlığını belirleyen etkenlerin faktör analizi ile incelenmesi”. Maliye Araştırmaları, 4, 61-83.
72. Wahyuningsih, R.D. and Hastjarjo, S. (2013). “Challenges in implementation of ICT for budget accountability and development of budget information literacy”. Bisnis and Birokrasi, 20(1), 51. DOI: 10.20476/jbb.v20i1.1868
73. Wijaya, T., Darmawati, A. and Kuncoro, A.M. (2020). "E-lifestyle confirmatory of consumer generation Z”. International Journal of Advanced Computer Science and Applications, 11(10), [http://dx.doi.org/10.14569/IJACSA.2020.0111004](https://dx.doi.org/10.14569/IJACSA.2020.0111004)
74. Wu, Q. and Qiu, Y. (2019). “Research on the Influencing Factors of China’s Logistics Industry Based o and Multiple Regression Model”. 2019 International Conference on Economic Management and Model Engineering (ICEMME), 360–363.
75. Xue, W. and Sun, S. (2019). “Relationship between organizational improvisation and organizational creativity under multiple regression analysis”. Revista de Cercetare Si Interventie Sociala, 65, 206–229.
76. The United Nations (UN) (2015). Transforming Our World: 2030 Agenda for Sustainable Development. Accessed 20 September 2024, https://sdgs.un.org/2030agenda
77. Yalçın, M. and Karaman, S. (2022). "An evaluation of the touristic product preferences of Generation Z individuals". Journal of Current Tourism Research, 6(2), 62–76.
78. Yildiz, F. and Alkan, F. (2017). “The research of perception budget of higher education students from participatory budgeting perspective: A case study of Dumlupinar University”. Research Journal of Public Finance, 3 (2), 129-14.
79. Yuan, H. (2015). “How financial literacy affects budgeting behaviours. Student scholarship - school of business”. Paper 4. Accessed 20 September 2024, [http://digitalcommons.georgefox.edu/gfsb_student/4.](http://digitalcommons.georgefox.edu/gfsb_student/4)
80. Yusuf, J.E., Jordan, M.M., Neill, K.A. and Hackbart, M. (2013). “For the people: Popular financial reporting practices of local governments”. Public Budgeting and Finance, 33(1), 95- 113.
