# Supplementary material for: Decoding budget awareness: A multivariate analysis of generation Z undergraduates
Source: PLoS One. 2025 Aug 14;20(8):e0328742. doi: 10.1371/journal.pone.0328742 (PMC12352852; doi:10.1371/journal.pone.0328742)
Supplement: Attachment — Submitted filename: Review of Decoding Budget Awareness_2003-2025.docx [file pone.0328742.s003.docx]

**RESULT OF REVIEW**

Title: *Decoding Budget Awareness: A Multivariate Analysis of Generation Z Undergraduates*

COMMENTS FROM REVIEWER

1. Once again, I would like to express my appreciation to the author who has diligently revised the paper by referring to several notes from the reviewer.
2. Article structure:

The author has improved the structure of the paper well, so the core of the paper, which includes The Results and Discussion, Conclusion, and Recommendations, has increased from 2,749 words to 3,800 words.

3. Originality

Some feedback has been accommodated and implemented by the author.

4. Abstract

Some feedback has been accommodated and implemented by the author.

5. Utilities of literature

The references have been enriched with more precise articles and their publications are quite recent.

6. Methodology

The methodology has been adjusted based on feedback from the reviewers.

7. Results and Discussions

This section has also been revised according to the suggestions and feedback from the reviewers.

8. Conclusion

The formulation of the conclusion is quite good, taking into account the notes and input from the reviewers.

9. Implications

The formulation in this section is already good.

10. Quality of Communication

The paper's sections have been well-written by the author.
