# Supplementary material for: Decoding budget awareness: A multivariate analysis of generation Z undergraduates
Source: PLoS One. 2025 Aug 14;20(8):e0328742. doi: 10.1371/journal.pone.0328742 (PMC12352852; doi:10.1371/journal.pone.0328742)
Supplement: Attachment — Submitted filename: Rebuttal Letter 07.04.2025.docx [file pone.0328742.s005.docx]

Dear Editor,

We sincerely appreciate the detailed and constructive feedback provided by you and the reviewers on our manuscript entitled *"*Decoding Budget Awareness: A Multiple Analysis of Generation Z Undergraduates*"* (Manuscript ID PONE-D-25-02233R1). We are grateful for the time you have dedicated to reviewing our work and for the valuable insights you have shared. We hope that we have responded to the comments to your satisfaction. In the original version, the requested revisions are highlighted in yellow.

Best regards

Authors

1. Reference should be more add and need more than 8 more references and prefer from the PLOS One Journal;

**Response:** To meet the requirement, we conducted a thorough search of PLOS One and added 11 relevant references from this journal to enhance the credibility and depth of our discussion.

2. Discussion must be enrich and add more references to support the discussion session; and

**Response:** We enriched the discussion section by incorporating additional references to strengthen our arguments. The newly added contributions are highlighted in yellow for clarity.

3. Each hypothesis should have minimum a paragraph for elaborating the idea in discussion session as a analysis.

**Response:** Each hypothesis was addressed in a dedicated paragraph where applicable. Due to the conceptual overlap between certain hypotheses, such as D3 and D5, they were jointly analyzed to ensure coherence and avoid redundancy.
