# Supplementary material for: Decoding budget awareness: A multivariate analysis of generation Z undergraduates
Source: PLoS One. 2025 Aug 14;20(8):e0328742. doi: 10.1371/journal.pone.0328742 (PMC12352852; doi:10.1371/journal.pone.0328742)
Supplement: Attachment — Submitted filename: Rebuttal Letter 02.06.2025.docx [file pone.0328742.s006.docx]

Dear Editor,

We sincerely appreciate the detailed and constructive feedback provided by you and the reviewers on our manuscript entitled *"*Decoding Budget Awareness: A Multiple Analysis of Generation Z Undergraduates*"* (Manuscript ID PONE-D-25-02233R2). We are grateful for the time you have dedicated to reviewing our work and for the valuable insights you have shared. We hope that we have responded to the comments to your satisfaction. In the original version, the requested revisions are highlighted in yellow.

Best regards

Authors

**Reviewer #1: This article has done to address the main point purpose of the article, but this article need to add more references.**

Dear Reviewer,

After your revision request, we have enhanced the manuscripts with new references. Now, the reference number has reached a total of 118 in its last version. By doing so, the paper has significantly improved in the introduction and especially the literature review parts. We have coloured the new contributions yellow. We thank you for reviewing our manuscript again.
